# Anti-Protozoan Activities of Polar Fish-Derived Polyalanine Synthetic Peptides

**DOI:** 10.3390/md21080434

**Published:** 2023-07-31

**Authors:** Ellynes Amancio Correia Nunes, Maria Cláudia da Silva, Marlon Henrique Cardoso, Sergio Leandro Espíndola Preza, Lucas Silva de Oliveira, Breno Emanuel Farias Frihling, Sébastien Olivier Charneau, Philippe Grellier, Octávio Luiz Franco, Ludovico Migliolo

**Affiliations:** 1 Postgraduate Program in Biochemistry, Federal University of Rio Grande do Norte, Natal 59078-900, Brazil; ellynesnunes@gmail.com; 2 S-Inova Biotech, Graduate Program in Biotechnology, Dom Bosco Catholic University, Campo Grande 79117-900, Brazil; mariaclaudiasilva@usp.br (M.C.d.S.); marlonhenrique6@gmail.com (M.H.C.); dyrosha@gmail.com (S.L.E.P.); brenoemanuelfarias@gmail.com (B.E.F.F.); ocfranco@gmail.com (O.L.F.); 3 Department of Biochemistry and Immunology, Faculty of Medicine of Ribeirão Preto, University of São Paulo, Sao Paulo 14040-900, Brazil; 4 Laboratory of Research in Virology and Immunology, Institute of Biological and Health Sciences, Federal University of Alagoas, Maceio 57020-600, Brazil; 5 Center for Proteomics and Biochemical Analysis, Catholic University of Brasília, Brasilia 71966-700, Brazil; 6 Laboratory of Biochemistry and Protein Chemistry, Department of Cell Biology, Institute of Biological Sciences, University of Brasília, Brasilia 73345-010, Brazil; lucassoliveira@outlook.fr (L.S.d.O.); charneau@unb.br (S.O.C.); 7 UMR 7245 Molécules de Communication et Adaptation des Micro-Organismes, Muséum National d’Histoire Naturelle, CNRS, 75005 Paris, France; philippe.grellier@mnhn.fr; 8 Graduate Program in Molecular Pathology, University of Brasilia, Brasilia 73345-010, Brazil; 9 Postgraduate Program in Cellular and Molecular Biology, Federal University of Paraíba, João Pessoa 58051-900, Brazil

**Keywords:** antimicrobial peptide, polyalanine, molecular dynamics, trypanocidal peptide, antiplasmodial peptide

## Abstract

Chagas disease, sleeping sickness and malaria are infectious diseases caused by protozoan parasites that kill millions of people worldwide. Here, we performed in vitro assays of *Pa-MAP*, *Pa-MAP1.9*, and *Pa-MAP2* synthetic polyalanine peptides derived from the polar fish *Pleuronectes americanus* toward *Trypanosoma cruzi*, *T. brucei gambiense* and *Plasmodium falciparum* activities. We demonstrated that the peptides *Pa-MAP1.9* and *Pa-MAP2* were effective to inhibit *T. brucei* growth. In addition, structural analyses using molecular dynamics (MD) studies showed that *Pa-MAP2* penetrates deeper into the membrane and interacts more with phospholipids than *Pa-MAP1.9*, corroborating the previous in vitro results showing that *Pa-MAP1.9* acts within the cell, while *Pa-MAP2* acts via membrane lysis. In conclusion, polyalanine *Pa-MAP1.9* and *Pa-MAP2* presented activity against bloodstream forms of *T. b. gambiense*, thus encouraging further studies on the application of these peptides as a treatment for sleeping sickness.

## 1. Introduction

Neglected tropical diseases (NTDs) are defined as a group of viral, bacterial and eukaryotic parasitic diseases commonly reported in low-income populations worldwide [1]. Among the NTDs transmitted by protozoan parasites from the genus *Trypanosoma*, two different types of diseases are reported in the vast majority of cases: (i) Chagas disease or American trypanosomiasis transmitted by *T. cruzi* [2] and (ii) sleeping sickness or African trypanosomiasis caused by infections by *T. b. gambiense* and *rhodesiense* [3].

Chagas disease affects 6–7 million people worldwide, mainly in Latin American countries in which the disease is endemic but also in non-endemic countries due to spreading by migration, such as Australia, Canada, Japan, Spain and the United States of America [4]. Although benznidazole, the standard drug used for *T. cruzi* infection [5], has been effective in eliminating the trypomastigote blood form, however, this drug presents intense side effects [6]. In addition, the efficacy of benznidazole decreases drastically if the treatment is started during the chronic phase of the disease instead of during the acute phase. Furthermore, certain *T. cruzi* strains are resistant to benznidazole treatment, thus representing an additional challenge in the clinical application of this drug [7]. While Chagas disease mainly occurs in Latin America, sleeping sickness is endemic in African countries where ~70 million people are at risk for contracting the disease. In the latest phase of the disease, *T. brucei* can cross the blood-brain barrier and then migrate to the central nervous system. In this context, the drugs capable of reaching and eliminating these parasites present high levels of toxicity and complex administration [8].

Recently removed from the list of NTDs, but no less devastating, malaria affects more than 220 million people worldwide and kills more than 400,000 people each year [9]. Moreover, malarial infections may be heading towards an even worse phase due to antimalarial drug resistance, mainly observed during *P. falciparum* infections [9,10]. Therefore, considering the alarming scenario imposed by *T. cruzi*, *T. brucei* and *P. falciparum*, the development of alternative drugs capable of acting against these pathogens and with low side effects on human cells is urgently required.

Bioinspired peptides and proteins have multiple pharmacological applications [11,12]. In the last few years, studies have characterized polyalanine peptides as multifunctional molecules with promising pharmacological potential [12,13,14]. Among them, we highlight the synthetic peptides *Pa-MAP*, *Pa-MAP1.9* and *Pa-MAP2*, which are derived from the polar fish *P. americanus*. In previous studies, *Pa-MAP* revealed antibacterial, antifungal, antiviral and antitumoral activities from 60 to 115 µM [13]. Moreover, *Pa-MAP2* has revealed promising antibacterial activity against Gram-negative strains both in vitro and in vivo [12].

Taken together, these findings emphasize the multifunctionality of polyalanine peptides derived from *P. americanus*, thus encouraging further biological studies in the context of NTDs. We therefore presented the evaluation of *Pa-MAP*, *Pa-MAP1.9* and *Pa-MAP2* trypanocidal and antiplasmodial activities, along with atomic level studies on how these peptides interact with a phospholipid bilayer mimicking the *Trypanosoma* membrane.

## 2. Results

### 2.1. Peptide Design Strategy

The start to modification, the peptides were the physic-chemical characteristics such as charge and hydrophobicity [15]. The first generation of the peptides focused on punctual substitutions; the second generation of the peptides focused on physic-chemical characteristics re-organized using helical wheels. The modifications in the second generation inspired a scaffold antifreeze peptide reflected by the increased activity [13,14,16].

Through the rational design, the *PaMAP* (HTASDAAAAAALTAANAAAAAAASMA) peptide was designed, and from that, two more models were designed, aiming to improve the observed multiactivity. *Pa-MAP1.9* (LAAKLTKAATKLTAALTKLAAAL) and *Pa-MAP2* (HKASDAAAKAALKAAKAAAKAAASMAK) had their loads increased by +4 and +6, respectively, maintaining the hydrophobicity of 60% to 80% and reorganizing the amphipathicity [13,14]. The three peptides were very active against various microorganisms and tumor cells.

Furthermore, the peptides were previously subjected to experiments to better understand their secondary and 3D structures, such as circular dichroism spectroscopy and molecular dynamics [13,14].

### 2.2. Anti-Protozoan Activities of Peptides

Remarkably, none of the three polyalanine synthetic peptides in this study showed hemolysis activity on red blood cells during the highest concentration test of 100 µg.mL^−1^. All the peptides were then tested for their activity against the chloroquine-resistant (CQ) K1 strain of *P. falciparum* (CQ: IC_50_ approximately 0.18 µM) [17]. Moreover, none of them were capable of inhibiting *P. falciparum* growth at lower doses than the cytotoxic dose on the mammalian myoblast derived L6 cell line (Table 1).

Similar to what was reported here was also observed regarding the anti-*T. cruzi* activity of the three polyalanine peptides tested. The concentration at which *Pa-MAP*, *Pa-MAP1.9* and *Pa-MAP2* inhibited *T. cruzi* growth was almost the same as the cytotoxic concentration on the mammalian cell line (Table 1).

### 2.3. Molecular Dynamics Structural Analyses

**Figure 1:**
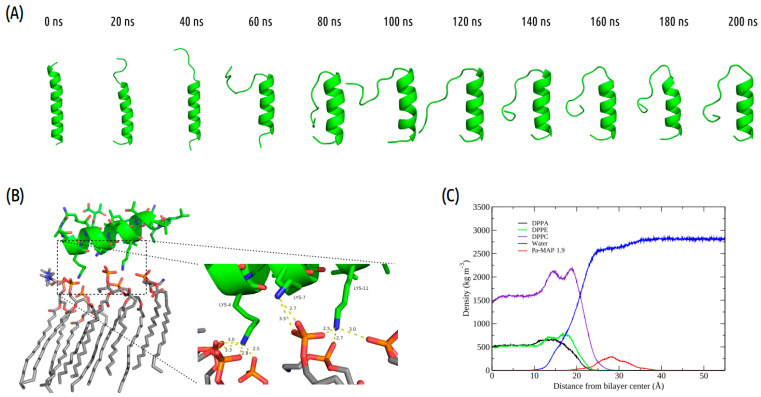
*Pa-MAP1.9* MD simulation in the membrane environment. (**A**) Structural snapshots during 200 ns of trajectory. (**B**) Tridimensional representation of *Pa-MAP1.9* (green), highlighting (dotted square) the region where there is an interaction between the nitrogen NZ of Lys^4^, Lys^7^ and Lys^11^ with the membrane lipids (orange) at 200 ns. (**C**) Mass density profile of *Pa-MAP1.9*, water and phospholipids during the last 5 ns of the simulation.

**Figure 2:**
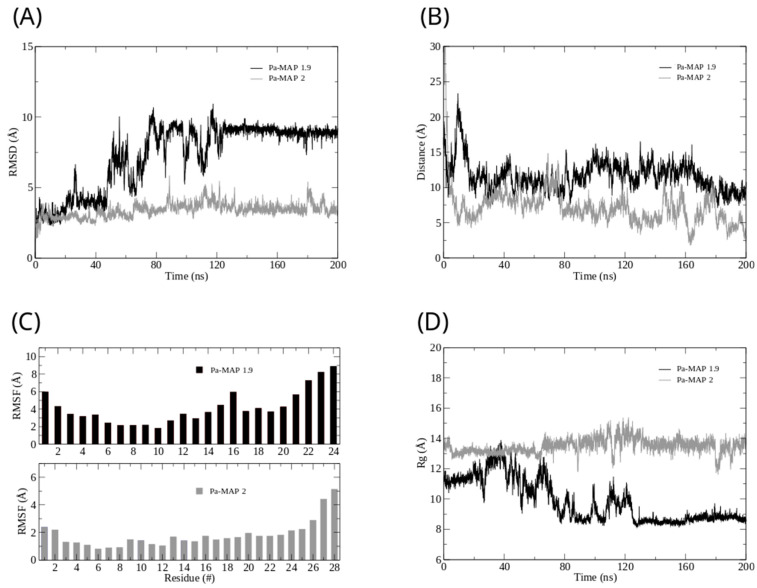
Root mean square deviation (**A**) for *Pa-MAP1.9* (black) and *Pa-MAP2* (gray). (**B**) Distance from the center of mass of *Pa-MAP1.9* (black) and *Pa-MAP2* (gray) during the simulation. (**C**) Root mean square fluctuation by residues of the peptides during MD. (**D**) Radius of gyration for *Pa-MAP1.9* (black) and *Pa-MAP2* (gray) during 200 ns of the simulation.

Interactions between *Pa-MAP1.9* or *Pa-MAP2* and a trypomastigote model membrane were evaluated by MD simulations. The time evolution of *Pa-MAP1.9* conformation during the 200 ns is shown in Figure 1A. After equilibration of the system, the peptide is located near the surface of the membrane. From there, we monitored the variation in the distance between the peptide’s center of mass and the membrane surface. As a result, we observed greater distance stability for *Pa-MAP1.9*, which at the end of the simulation was located approximately 10 Å from the membrane surface (Figure 2B).

Furthermore, we analyzed the *z*-axis density profile for the *Pa-MAP1.9* system, considering zero as the center of the membrane during the last 5 ns of the MD simulation (Figure 1C). We observed that *Pa-MAP1.9* is located just above the interface between the membrane and water, theoretically suggesting that this peptide interacted with but did not penetrate the membrane. The RMSD for this peptide indicated the existence of two distinct states. From the initial state to ~130 ns, there was a larger deviation in the peptide trajectory. From this point on, the peptide reached a more stable conformation that was preserved until the end of the 200 ns of MD (Figure 2A).

This same pattern appears when the radius of gyration is analyzed (Figure 2D). It is possible to see that the size of the peptide is similar to the beginning of the simulation and then decreases. After 130 ns, the peptide finds a more stable conformation and remains in it until the end of the simulation, corroborating the behavior observed by the RMSD. The RMSF graph for *Pa-MAP1.9* (Figure 2C) indicates a large fluctuation of its residues, mainly those at the C-terminal region (residues 16LTKLAAALT24).

**Figure 3:**
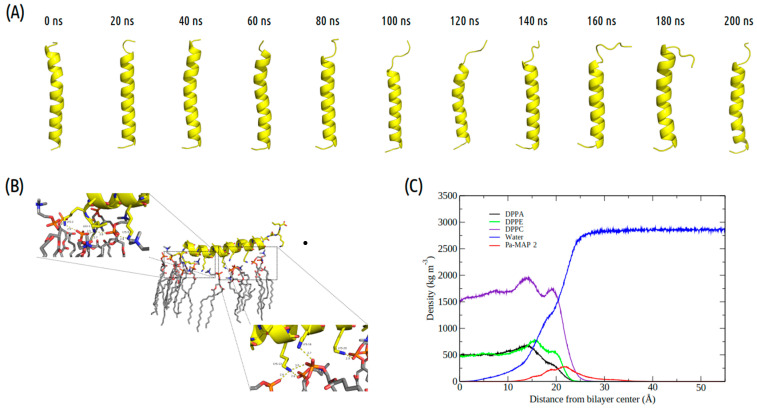
Evaluation of the molecular dynamics of *Pa-MAP2*. (**A**) Structures demonstrated during 200 ns of trajectory. (**B**) Tridimensional representation of the *Pa-MAP2* (yellow), highlighting (dotted squares) the region where there is interaction between the nitrogen NZ of Lys^2^, Lys^9^, Lys^13^, Lys^16^, Lys^20^ and Leu^1^ to membrane lipids (orange) at 200 ns. (**C**) Mass density profile of the *Pa-MAP2* peptide, water and lipids during the last 5 ns of simulation.

The time evolution of the *Pa-MAP2* structural conformation during the 200 ns in contact with the trypomastigote membrane model is shown in Figure 3A. Compared to *Pa-MAP1.9*, at 200 ns, *Pa-MAP2* is located closer to the membrane surface, as observed by the mass density profile (Figure 3C) and the membrane surface distance (Figure 2B). The peptide has embedded more deeply into the water/membrane interface, with lysine side chains interacting with the membrane and alanine side chains facing the solvent region.

It was possible to see a greater distance of stability for the peptide, and at the end of the simulation, it was located within approximately 5 Å of the surface of the membrane (Figure 2B). We show the density profile in the *z*-axis for the *Pa-MAP2* system, using zero as the center of the membrane during the last 5 ns of simulation (Figure 3C). This peptide is located below the interface between the membrane and water, lying on the surface of the bilayer. The RMSD (Figure 2A) indicates great stability of its 3D structure during the simulation, as the behavior confirmed by the radius of the gyration (Figure 2D) where there is no large variation in structure, remaining approximately the same size during the MD.

The RMSF graph for *Pa-MAP2* indicates a small fluctuation of its residues (Figure 2C), possibly due to its increased interaction with the membrane.

**Figure 4:**
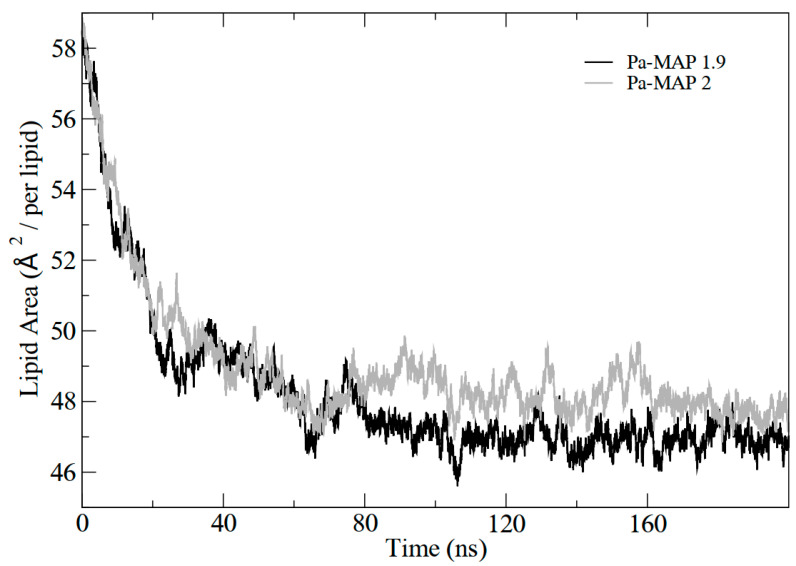
Lipid area graph during molecular dynamics for *Pa-MAP1.9*(black) and *Pa-MAP2* (gray) peptides.

**Figure 5:**
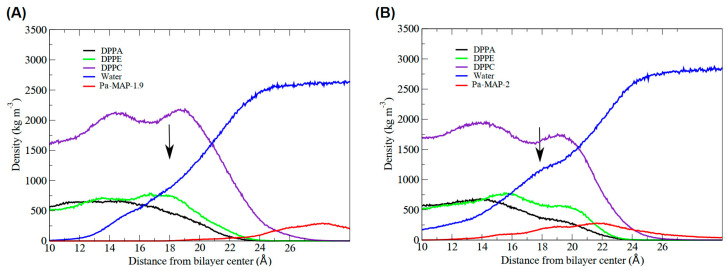
Highlight of the interface region of the mass density profile for the peptides. The black arrow points where, for (**A**) *Pa-MAP1.9* and (**B**) *Pa-MAP2*, the water density slightly increased, forming a shoulder-like, indicating permeabilization on the bilayer surface. The red curve shows the density of the peptides (approximation) in relation to the membrane.

In addition, the results demonstrated the variation in the area per lipid during the simulations, which can be seen in Figure 4. It indicates that the greater penetration of *Pa-MAP1.9* caused a slight decrease in the lipid area compared to *Pa-MAP2*. In Figure 5, the mass density profiles at the interface between the membrane and the water for the two peptides show that there is a small penetration of water on the membrane surface for the simulation of *Pa-MAP2*, which does not occur for the other peptide. This indicates that the disturbance caused by this peptide favors the internalization of water molecules.

## 3. Discussion

### 3.1. Peptides Design Strategy

Initially, synthetic polyalanine peptides were rationally designed based on the antifreeze peptide model of *P. americanus*, a polar fish [15]. The first generation was designed based on composition and hydrophobicity, which led to the development of *Pa-MAP* with the amino acid sequence HTASDAAAAAALTAANAAAAAAASMA, characterized as a multiactive peptide. It has been reported to exhibit antibacterial activity against *E. coli* and *S. aureus* strains, with a better MIC for *E. coli* (30 µM). Furthermore, it showed antiviral activity against herpes simplex viruses 1 and 2 (HSV-1 and HSV-2) with more than 90% inhibition for both viral types at a concentration of 90 µM. Antifungal and antitumor activity have also been reported, although with less significance against the yeast *C. parapsilosis*. The peptide demonstrated efficacy against the tested tumor cell lines (Caco-2 [human colorectal adenocarcinoma epithelial cells], HCT-116 [human colorectal carcinoma cell lines] and MCF-7 [human breast cancer cell]) [15].

To further enhance its activity, a second generation of Pa-MAP-based peptides were designed and identified as the most effective synthetic peptides against bacteria, biofilms and tumor cells. *Pa-MAP1.9* had its amino acid sequence modified to LAAKLTKAATKLTAALTKLAAALT, increasing the charge to +4 while maintaining 60% hydrophobicity. This modification improved the antibacterial activity against *E. coli*, decreasing the MIC from 30 to 6–12 µM. It also showed efficacy against other bacterial strains such as *Enterococcus faecalis* and *Klebsiella pneumoniae* [13,14]. Another variant, *Pa-MAP2*, underwent lysine substitutions in some amino acids, resulting in the sequence HKASDAAAKAALKAAKAAAKAAASMAK, with an increased charge of +6 and maintained hydrophobicity at 80%. This modification was even more effective against *E. coli* and *Acinetobacter baumannii* strains, with MIC values of 3.2 µM. Notably, for both peptides, the amphipathicity was rearranged and improved [13,14,15]. All three peptides exhibited potent activity against various microorganisms and tumor cells.

### 3.2. Anti-Protozoan Activities of Peptides

The results observed here indicate a poor pharmacological potential of these three peptides for inhibition of *P. falciparum* erythrocyte stages and the intracellular trypomastigote forms of *T. cruzi*.

Unlike our findings, a peptide synthesized based on the venom of the bee *Polybiapaulista*, polybia-CP (ILGTILGLLSKL-NH2) proved to be effective against *T. cruzi* strains in all forms tested, in addition to being active at non-toxic concentrations for mammals [18].

The same was observed for Motobamide, a peptide derived from a marine cyanobacteria, which was shown to be active against blood forms of *T. brucei rhodesiense* with an IC_50_ of 2.3 μM, with cytotoxicity observed at an IC_50_ of 55 μM [19].

Additionally, 3 peptides (kakeromamide B, ulongamide A and lyngbyabellin A) also isolated from a marine cyanobacteria are active against blood forms of *P. falciparum*, with an EC_50_ of 0.89, 0.99 and 0.15 µM, respectively [20].

Nevertheless, more promising results were obtained against *T. b. gambiense* bloodstream forms. Anti-*T. b. gambiense* activity was not observed for *Pa-MAP* at lower doses than the cytotoxic dose (45.2 µM) (Table 1). Nonetheless, both *Pa-MAP1.9* and *Pa-MAP2* inhibited *T. b. gambiense* growth at 1.2 and 5 µM (Table 1), while the cytotoxic doses were 26 and 46 µM, respectively. In previous works, it was observed that *Pa-MAP1.9* and *Pa-MAP2* had their activities improved, and the authors report that the change in the charge of the peptides, which before was −1 and passed to +4 and +6, were determining factors in the potentiation of the activity, as well as the insertion of hydrophilic residues in place of hydrophobic residues, thus changing the amphipathicity of the peptides. Similar results were observed here, since *Pa-MAP* only achieved its activity at toxic doses, but its derivatives had activity at concentrations below the toxic dose of the peptides [13,14,15].

These findings resulted in SI values of 8.1 for *Pa-MAP2* and 31.03 for *Pa-MAP1.9*, suggesting a high efficiency of the latter for sleeping sickness therapy. An important acceptance criterion for the development of a new drug is the SI value, which should be superior to ten [21]. Considering that SI is the toxic concentration (TC_50_) divided by the inhibitory concentration (IC_50_), a new drug should inhibit the parasite growth in a dose at least 10 times lower than the cytotoxic dose.

To the best of our knowledge, this is the first report of a polyalanine peptide (*Pa-MAP1.9*) with an SI above 20 in anti-*T. b. gambiense* in vitro biological assays. Previous studies, however, have already demonstrated that peptides belonging to the class of the cathelicidins, including novispirin, ovispirin, *SMAP-29* and protegrin-1, are effective in killing both the procyclic (insect form) and the human bloodstream forms of *T. brucei* spp. [22]. Moreover, studies have also reported the effectiveness of cell-penetrating peptides in reducing the viability of *T. b. gambiense* bloodstream forms by crossing the parasite membrane to act on intracellular targets [23].

In the search for new composts that are more efficient and do control infections caused by *T. cruzi*, a group of researchers tested ruthenium complexes based on rational developments, reducing toxicity and increasing water solubility. These compounds act by blocking the lipid biosynthesis pathway, which is essential for the parasite. In this way, was observed that the compound 1 was able to reduce the resistant trypomastigotas forms with an IC_50_ of 79 ± 3 µM in vitro, and the epimastigotas forms with an IC_50_ were 127 ± 8 µM [24]. In comparison, *Pa-MAP1.9* and *Pa-MAP2* have an IC_50_ still lower; nevertheless, our tests are still framed as initial tests, with new experiments necessary that contribute to the future development of new drugs inspired by the peptides tested here.

### 3.3. Molecular Dynamics Structural Analyses

The theoretical results provide evidence that the peptides have different behaviors in their interaction with the membrane, as shown by the experimental results. *Pa-MAP* and *Pa-MAP2* presented a hydrophobic amino acid composition above 70%, as well as lower (+1) and higher (+6) charges, indicating that the hydrophobicity and charge are determining parameters for antiprotozoan activity. This is because the *Pa-MAP1.9* was more active against *T. brucei*, presenting a moderate charge (+4) and hydrophobicity (64%), in comparison with our analogues and average antimicrobial peptides deposited in the Antimicrobial Database [25].

Taken together, these results give an idea of different initial behaviors when approaching the trypomastigote membrane. The fact that the structures are different indicates different modes of interaction. Nevertheless *Pa-MAP1.9* lost some of the initial α-helix conformation (from 87% to 50%) and did not disturb the membrane surface during the simulation. This result is interesting because, despite interacting with the membrane, it is expected that this interaction will not be as strong or disturbing, as the experimental results observed for this peptide is that it has its mechanism of action inside the cell [14]. Thus, it is important that it should cross the membrane, but without causing damage to its structure. When approaching the membrane, *Pa-MAP1.9* presents three lysine residues forming hydrogen bonds (HB) with oxygen atoms from the membrane phospholipids, possibly due to the low penetration in the bilayer and to losing part of its secondary structure (Table S1).

*Pa-MAP2*, on the other hand, varied little during the simulation, with 93% α-helix at the beginning of the simulation and 78% at the end, and it interacted more strongly with the membrane, lying on the surface and pushing water molecules to the interior of the bilayer. This peptide presents four lysine residues and one leucine residue participating in HB with the phospholipids, which attributed greater stability and moved this peptide closer to the membrane surface (Table S2). This result indicates that it behaves as previously mentioned for *T. brucei*, where it was characterized as an active peptide in the membrane trapped between the phospholipids [26].

## 4. Materials and Methods

### 4.1. Peptide Synthesis and Preparation

*Pa-MAP1.9* (NH2-LAAKLTKAATKLTAALTKLAAALT-COOH) and *Pa-MAP2* (NH2-LKAAAAAAKLAAKAAKAALKAAAAAAKL-COOH) were designed and synthesized by the Fmoc strategy, loosely based on the sequence of a previously described multifunctional peptide, *Pa-MAP* (NH2-HTASDAAAAAALTAANAAAAAAASMA-COOH) [27]. MALDI-ToF analysis showed greater than 95% purity and monoisotopic ions of 2667.6 and 2517.6 Da, in agreement with the theoretical calculated molecular mass obtained by Protein Prospector (http://prospector.ucsf.edu/prospector/mshome.htm (accessed on 22 June 2023)). Afterwards, the peptides were resuspended in purified water (milli-Q grade) at a stock concentration of 2 mg·mL^−1^ at a ratio of 1:1 (*w*:*v*), filtered with 0.22 μm pore filter membranes and stored at −80 °C.

### 4.2. In Vitro Bioassay of Antiplasmodial Activity

The chloroquine-resistant K1 strain of *P. falciparum* (CQ: IC_50_approximately0.18 µM) was maintained in vitro on human erythrocytes in RPMI 1640 medium supplemented with 8% (*v*:*v*) heat-inactivated human serum at 37 °C and under an atmosphere of 3% CO_2_, 6% O_2_ and 91% N_2_. An in vitro drug susceptibility assay was evaluated by [^3^H]-hypoxanthine incorporation as previously described. *Pa-MAP*, *Pa-MAP1.9* and *Pa-MAP2* peptides were serially diluted two-fold with 100 μL culture media in 96-well plates. All wells were completed with 100 μL ring enriched parasite cultures (1% parasitaemia and 1% final hematocrit) with the final peptide concentration ranging from 100 to 0.05 μg.mL^−1^ and incubated for 24 h at 37 °C. Prior to freezing and thawing the plates, a further incubation of 24 h occurred after the addition of 0.5 μCi of [3H]-hypoxanthine (GE Healthcare, France, 1 to 5 Ci·mmol·mL^−1^). Cell lysates were then collected onto glass-fiber filters and counted in a liquid scintillation spectrometer. The growth inhibition for each peptide concentration was determined by comparison of the radioactivity incorporated in the treated culture with that in the control culture maintained on the same plate. The concentrations inhibiting 50% of parasite growth (IC_50_) were determined by linear regression analysis from all synthetic peptides with a concentration-response curve, and the results were expressed as the mean values ± standard deviations determined from three independent experiments. Chloroquine prepared in purified water (milli-Q grade) at 10 mM was used as the positive control [28,29,30,31,32].

### 4.3. In Vitro Bioassay of Anti-Trypanosoma cruzi Activity

The β-galactosidase-expressing *T. cruzi* trypomastigotes (Tulahuen strain—*lacZ* clone 4) were maintained in L6 cell monolayers grown in RPMI medium supplemented with 10% (*v*:*v*) fetal calf serum at 37°C and 5% CO_2_. Inhibition assays of intracellular parasite multiplication were performed in 96-well plates as described previously. Briefly, a monolayer of 5 × 10^3^ L6 myoblasts was incubated with 5 × 10^4^ trypomastigotes per well for 18 h. Cells were then incubated with two-fold dilutions of each *Pa-MAP*, *Pa-MAP1.9* and *Pa-MAP2* peptide, ranging from 100 to 0.05 μg.mL^−1^ for 5 days. After addition of the substrate chlorophenolred-ß-D-galactopyranoside at a 100 μM final concentration and Nonidet P-40 (0.1% final concentration) (Buckner, Verlinde, La Flamme and Van Voorhis 1996), the plates were incubated for 4 h at 37 °C. The media turned from yellow to red due to ß-galactosidase activity and quantitated at 570 nm by an automated microplate reader spectrophotometer. IC_50_ values were obtained from the peptides’ concentration-response curve, and the results were expressed as the mean values ± standard deviations determined from three independent experiments. Nifurtimox prepared in DMSO at 10 mg·mL^−1^ was used as the positive drug control and DMSO as the negative control [30,31,33,34,35,36].

### 4.4. In Vitro Bioassay of Anti-Trypanosoma brucei gambiense Activity

Bloodstream forms of *T. b. gambiense* (strain Feo) were cultured in HMI9 medium supplemented with 10% fetal calf serum at 37°C and 5% CO_2_. In all experiments, log-phage cell cultures were harvested by centrifugation at 3000× *g* and immediately used. The peptide assays were based on the conversion of a redox-sensitive dye (resazurin sodium salt, SIGMA, Missouri, USA) to a fluorescent product by viable cells. The in vitro bioassay of anti-*T. b. gambiense* activity was performed as previously described [23,24,29]. Briefly, 1 × 10^4^ *T. b. gambiense* bloodstream forms were placed in 200 μL of culture medium per well either in the absence or in the presence of different peptide concentrations ranging from 100 to 0.05 μg.mL^−1^. After a 72h incubation period, 45 μM (final concentration) resazurin solution was added to each well and, after 4 h further incubation, fluorescence was measured at 530 nm excitation and 590 nm emission wavelengths. The percentage of inhibition of the parasite growth was calculated by comparing the fluorescence of parasites maintained in the presence or absence of peptide. IC_50_ values are the mean +/− the standard deviations. Pentamidine prepared in DMSO at 10 mM was used as the anti-trypanosomal drug control [30,31,37,38,39].

### 4.5. Cytotoxic Activity on L6 Myoblasts

Monolayers of L6 myoblasts at 5 × 10^3^ cell per well of 96-well plates in 200µL of RPMI medium containing 10% fetal calf serum were maintained with different peptide concentrations ranging from 100 to 0.05 μg.mL^−1^ for 5 days at 37 °C under a 5% CO_2_ atmosphere. Cytotoxicity was determined using the colorimetric MTT assay, and the absorbance reduction percentages at 540 nm for the treated cultures and the untreated control cultures were obtained and compared. The concentrations causing 50% of cell growth inhibition (TC_50_) were obtained from the peptides’ concentration response curves. The results were expressed as the mean values ± standard deviations determined from three independent experiments [31,40].

### 4.6. In Vitro Hemolysis Assay

The three peptides were serially diluted with the culture medium (RPMI + 8% human serum) in 96-well plates, and the same medium containing human erythrocytes was added to each well for a 1% final hematocrit. After 48 h at 37 °C under a CO_2_ atmosphere, the hemoglobin absorbance at 540 nm was measured to define the hemolysis percentage. The absorbance measured in totally lysed erythrocyte culture by freezing/thawing was defined at 0% of lysis. Final concentrations of the peptides ranged from 100 to 0.05 μg.mL^−1^ [31].

### 4.7. In Silico Studies with a Trypomastigote Membrane

The trypomastigote membrane was built by the web-based graphical user interface online server CHARMM-GUI, using the Membrane Builder module. The trypomastigote bilayer model constructed (3:1:1) contains 60% phosphatidylcholine (PC), 20% phosphatidyl ethanolamine (PE) and 20% phosphatidic acid (PA). All peptides were placed 40 Å from the membrane surface, solvated with water molecules in the TIP3P model and chlorine ions added to neutralize the system [41,42].

### 4.8. In Silico Structural Analysis

Initially, the tridimensional theoretical structures for *Pa-MAP1.9* and *Pa-MAP2* were obtained by comparative modeling using the crystal structure of an antifreeze peptide (PDB: 1wfa) as the template. Molecular modeling simulations were performed on Modeller v.9.12 by satisfaction of spatial restraints. A total of 200 models were generated, and those with the lowest free energy (DOPE score) for *Pa-MAP1.9* and *Pa-MAP2* were selected for validation procedures using ProSa-web and PROCHECK. Molecular dynamics (MD) simulations were carried out using the Amber software, version 16. Usual parameters taken from the ff14SB force field were used to model all the peptides and phospholipids. The TIP3Pwater model was used in all simulations. During the simulations, phospholipids and solvent (counterions and water) were maintained at a constant temperature of 310 K using the Langevin thermostat with the collision frequency of 1.0 ps^−1^. These temperatures were chosen as they are above the gel-to-liquid crystal phase transition temperatures of all the lipids used in the simulations. The Van der Waals interactions were truncated at 1.2 nm with a long-range dispersion correction applied to the energy and pressure. A pressure of 1 bar was maintained using semi-isotropic pressure coupling with the Monte Carlo barostat and a time constant of 1 ps. Electrostatic interactions were treated using the smooth particle mesh Ewald (PME) algorithm with a short-range cutoff of 1.2 nm. The neighbor list was updated every 25 steps during the simulations. All bonds involving hydrogen are constrained using the SHAKE algorithm allowing a 2fs time step to be applied for 200 ns of MD simulation. All membrane/peptide systems were neutralized with Na^+^ ions. MD simulations were analyzed by means of root mean square fluctuation (RMSF), root mean square deviation (RMSD), mass density profile and distance to the plane of the membrane surface [43,44,45,46].

## 5. Conclusions

Overall, to the best of our knowledge, here we reported the first study with polyalanine peptides (*Pa-MAP2* and mainly *Pa-MAP1.9*) capable of controlling *T. b. gambiense* growth in vitro.

The simulations reinforce the importance of in silico strategies in elucidating the mechanisms of action for the studied peptides. The theoretical results provideevidence of the different behavior between the two peptides when approaching the membrane surface, showing a possible difference between the interaction and action mechanisms in the trypomastigote cell. Future work, with more simulation time, may clarify how each of them interact with the protozoan membrane and investigate whether treatments with both peptides are efficient at eliminating the parasite in infected mice. In summary, in this study, we show two new candidates for the treatment of sleeping sickness.

## Figures and Tables

**Table 1:** Antiplasmodial and anti-trypanosomal activities of *Pa-MAP*, *Pa-MAP1.9* and *Pa-MAP2* peptides.

Peptide	Anti-Plasmodial Activity		Anti-*T. cruzi * Activity		Anti-*T. bruceigambiense * Activity		Cytotoxicity against L6 Cell
	(µM)						
	IC_50_ ± SD	SI	IC_50_ ± SD	SI	IC_50_ ± SD	SI	TC_50_ ± SD
*Pa-MAP*	>45.2	1.0	>45.2	1.0	>45.2	1.0	>45.2
*Pa-MAP1.9*	35.0 ± 1.2	1.1	>37.2	1.0	1.2 ± 0.1	31.0	>37.2
*Pa-MAP2*	47.0 ± 6.9	>1	>46.0	1.0	5.7 ± 1.2	>8.1	>46.0
Chloroquine	0.2 ± 0.1	>1887					>434.8
Nifurtimox			0.6 ± 0.1	35.9			22.3 ± 8.0
Pentamidine					0.004 ± 0.000	25,000	>100
DMSO	<2%: *w*/*o* significant effect						

The Chloroquine-resistant *P. falciparum* strain K1.Bloodstream forms of *Trypanosoma brucei gambiense* strain Feo.The β-galactosidase-expressing *Trypanosoma cruzi* trypomastigotes of the Tulahuen strain.Values of IC_50_ (concentrations inhibiting 50% of parasite growth) are represented by means of three replicates± standard deviations (SD).Selectivity indexes (SI) were determined by the ratio of cytotoxicity to biological activity (TC_50_/IC_50_).

## Data Availability

Not applicable.

## Supplementary Materials

The following supporting information can be downloaded at: https://www.mdpi.com/article/10.3390/md21080434/s1, Table S1: Lipid and amino acid position and their respective atom names and interaction length for *Pa-MAP1.9*; Table S2: Lipid and amino acid position and their respective atom names and interaction length for *Pa-MAP2*.
