# Supplementary material for: Anti-Protozoan Activities of Polar Fish-Derived Polyalanine Synthetic Peptides
Source: Mar Drugs. 2023 Jul 31;21(8):434. doi: 10.3390/md21080434 (PMC10456387; doi:10.3390/md21080434)
Supplement: Supplementary file 1 [file marinedrugs-21-00434-s001.zip › marinedrugs-2494006-supplementary.pdf]

Supplementary Material: Anti-protozoan activities of polar fish-derived polyalanine synthetic peptides

Table S1. Lipid and amino acid position and their respective atom names and interaction length for *Pa-MAP* 1.9.

Lipid	Atom	Length (Å)	Atom	Amino Acid	Interaction
DPPE^{96}	O13	2.7	NZ	LYS^{11}	SB
DPPA^{86}	O14	2.5	NZ	LYS^{11}	SB
DPPE^{81}	O13	3.0	NZ	LYS^{11}	SB
DPPA^{86}	O12	3.5	NZ	LYS^{7}	SB
DPPA^{86}	O13	2.7	NZ	LYS^{7}	SB
DPPC^{75}	O14	2.8	NZ	LYS^{4}	SB
DPPA^{89}	O13	2.5	NZ	LYS^{4}	SB
DPPC^{70}	O13	3.0	NZ	LYS^{4}	SB
DPPC^{75}	O12	3.3	NZ	LYS^{4}	SB

SB: Saline Bond

Table S2. Lipid and amino acid position and their respective atom names and interaction length for *Pa-MAP* 2.

Lipid	Atom	Length (Å)	Atom	Amino Acid	Interaction
DPPE^{17}	O14	2.9	NZ	LYS^{20}	SB
DPPC^{69}	O13	2.7	NZ	LYS^{16}	SB
DPPC^{36}	O14	2.6	NZ	LYS^{13}	SB
DPPC^{50}	O13	2.6	NZ	LYS^{13}	SB
DPPC^{70}	O14	2.8	NZ	LYS^{9}	SB
DPPC^{70}	O13	2.6	N	LEU^{1}	HB
DPPA^{73}	O14	2.5	N	LEU^{1}	HB
DPPC^{37}	O13	2.7	N	LUE^{1}	HB

SB: Saline Bond
HB: Hydrogen Bond
